# Factors Associated with Perceived Teacher-Related Change in Academic Resilience: Classroom Motivational Climate, Subjective Resilience, and Success Expectancies

**DOI:** 10.3390/bs16091526

**Published:** 2026-08-29

**Authors:** Mercedes Villasana Terradillos, Jesús Alonso-Tapia

**Affiliations:** 1 Facultad de Ciencias Humanas y Sociales, Universidad Pontificia Comillas, C/Universidad Comillas, 3-5, 28049 Madrid, Spain; 2 Facultad de Psicología, Universidad Autónoma de Madrid, C/Ivan Pavlov, 6, 28049 Madrid, Spain; jesus.alonso@uam.es

**Keywords:** academic resilience, classroom motivational climate, success expectancies, teacher-related change, perceived change, adolescence

## Abstract

Research on academic resilience has often focused on students’ personal resources, but less attention has been paid to those classroom conditions that may help students cope with academic setbacks. This study examined the extent to which students’ perceptions of a learning-oriented classroom motivational climate (CMC) were associated with perceived teacher-related change in academic resilience after accounting for subjective resilience and success expectancies. A total of 749 students attending a school in France in secondary, upper-secondary, and vocational education completed measures of academic resilience, CMC, success expectancies, and perceived change in resilience attributed to their teacher’s work. Using cross-sectional data, a path analysis with latent variables (PALVs), followed by cross-validation, supported the proposed model. CMC showed the strongest direct association with perceived change in academic resilience, whereas subjective resilience showed only a small association and success expectancies were not directly associated with perceived change. However, success expectancies were related to students’ perceptions of CMC and have a small indirect effect on perceived change in resilience. These results contribute to research on classroom motivational climate by identifying an association between CMC and students’ perceived teacher-related change in academic resilience after accounting for subjective resilience and success expectancies.

## 1. Introduction

In school or at home, adolescents have to cope not only with the stress generated by study pressures and setbacks in relation to their academic work, but also with stress arising from teachers’, peers’, and family members’ attitudes towards them. When faced with such adversities, whether minor or major, some adolescents experience considerable difficulty, whereas others adapt positively or recover and are therefore considered academically resilient (A. J. Martin & Marsh, 2009). Contemporary developmental accounts define resilience as adaptive functioning within dynamic systems that draw on personal, relational, and contextual resources rather than as a fixed individual attribute (Masten et al., 2021, 2023). From a positive youth development perspective, resilience can also be understood as emerging through mutually influential relations between adolescents and their developmental contexts (Lerner et al., 2023). Current reviews of resilience measurement also stress that resilience is expressed through promotive and protective factors operating across individual, relational, communal, and cultural levels, and that assessment should remain sensitive to context (Terrana & Al-Delaimy, 2023). Prevention-oriented models of adolescent resilience similarly distinguish personal assets and contextual resources as modifiable promotive factors (Zimmerman et al., 2013). Although academic resilience has been operationalized in different ways (Rudd et al., 2021), a recent review of protective factors identified the relevance of both personal and school-related factors and showed that the associations found vary to some extent depending on how resilience is operationalized, the data sources, and the research methods employed (Ye et al., 2024). In addition, meta-analytic evidence indicates that both risk and protective factors are significantly associated with achievement outcomes in academic resilience research. However, the average associations are modest. This fact reinforces the need to examine specific, modifiable school-related resources rather than assuming that resilience is explained only by students’ prior characteristics (Hunsu et al., 2023). These considerations raise two important questions: (1) On which psychological factors do academic resilience depend? and (2) How could educators, especially teachers and parents, support academic resilience?

This study addresses the second question by focusing on the school environment and examining students’ perceptions of teachers’ contributions to changes in their academic resilience. However, it is first necessary to identify the psychological factors that contribute to students’ resilience or lack thereof, especially those related to adversities experienced at school. This knowledge would provide a basis for looking into classrooms in search of teaching patterns that may contribute to enhancing resilience. Key insights into the psychological factors associated with resilience in academic contexts come from research on motivation and coping. Hence, they are considered next.

Motivation and resilience. Although achievement motivation is important for students’ academic success, motivation alone is not sufficient to ensure success. They also need to be able to deal with setbacks, study pressure, and stress generated by academic activities and teachers’ and peers’ attitudes, that is, they need to recover from adversities in order to be resilient (A. Martin, 2002; Yeager & Dweck, 2012). More recent longitudinal research has shown that academic buoyancy, a related capacity to manage everyday academic setbacks, predicted lower subsequent academic adversity (A. J. Martin & Marsh, 2020). Recent evidence also links teacher support and academic self-efficacy to dimensions of academic motivational resilience, suggesting that students’ resilient responses to academic challenges are connected to both personal motivational beliefs and teacher-related resources (Kang et al., 2024). Motivation theories provide important clues as to why some students may lack resilience despite being highly motivated, whereas others recover successfully from adversity.

On the one hand, students with a high fear of failure (Atkinson, 1957), a tendency to attribute failures to factors beyond their control (Weiner, 1986), or low self-efficacy and control expectancies (Bandura, 1997) tend to respond to academic and social adversities with cognitive and self-regulatory processes that hinder effective coping. The same applies to students who are avoidance-oriented rather than learning-oriented (Good & Dweck, 2006; Linnenbrink-García et al., 2012), or state-oriented rather than action-oriented (Kuhl, 1994), or to students who hold an entity theory of intelligence (Yeager & Dweck, 2012). Their expectations and thought processes when addressing a task, their focus on difficulties and on the possibility of failure, and their tendency to attribute academic and social failures to a lack of ability and control may hinder recovery from adversity and thereby undermine resilience.

On the other hand, students with a high need for achievement—who attribute failures to factors that they can control, believe that they can learn how to overcome failures and cope with difficulties, and are mastery-oriented rather than avoidance-oriented—tend to confront setbacks, study pressure, and stress in a completely different way. From this perspective, these students tend to consider how they may overcome difficulties. They also tend to focus on actions aimed at solving problems rather than on the emotional responses elicited by adversity. Finally, they tend to focus on the strategies they use when approaching a task rather than on the difficulties they face. More recent motivational theory emphasizes that success expectancies develop through students’ interpretations of prior performance and messages received from different socializers within particular cultural and educational contexts (Eccles & Wigfield, 2020). Recent integrative work similarly conceptualizes motivational resilience as patterns of action that enable students to deal constructively with, recover from, and learn from academic obstacles and failures (Skinner et al., 2020).

In light of the above evidence, it can be hypothesized that teaching patterns aimed at enhancing students’ motivation and promoting changes in students’ self-beliefs and expectations, as well as in their attitudes towards learning, effort, and strategy use, may be positively associated with resilience.

Coping and resilience. From current systems perspectives, resilience cannot be reduced to a stable personality factor but involves dynamic regulatory processes through which students interpret, manage, and respond to adversity (Masten et al., 2021; Terrana & Al-Delaimy, 2023). In this sense, coping remains relevant because it directs attention to the strategies students use when they face academic or relational stress. Research on coping has distinguished responses oriented towards problem solving, adaptive regulation, and support seeking from less-adaptive responses centered on avoidance, rumination, or emotional dysregulation (Lazarus & Folkman, 1984; Skinner et al., 2003; Skinner & Zimmer-Gembeck, 2007). Previous work with adolescent students showed that coping strategies accounted for a substantial proportion of resilience, and that personality factors added little once coping processes were considered (Villasana et al., 2016, 2017). This is consistent with recent views of academic motivational resilience not as a fixed individual disposition but as a set of action patterns—including reflection, help-seeking, perseverance, and emotional regulation—that can be supported by educational contexts (Skinner et al., 2020).

Coping strategies involve a focus on processes. Hence, as in the case of motivation, it can be hypothesized that teaching patterns that foster cognitive and emotional self-regulation in response to academic and social adversities may be positively associated with resilience. Which teaching patterns, then, may support mastery motivation, positive cognitive and emotional self-regulation, and resilience?

Classroom motivational climate (CMC). Since Ames (1992) introduced the concept of CMC, researchers have gathered evidence on teaching patterns associated with students’ motivation to learn (Meece et al., 2006). Ames (1992) proposed that CMC could foster mastery- or performance-goal orientations depending on teachers’ practices in six teaching areas represented by the acronym TARGET: task, authority, recognition, grouping, evaluation, and time. She proposed that specific teaching patterns in each of the areas could foster mastery orientation, whereas their absence or the use of opposing patterns could impede it and promote performance-oriented goals. Much of the research on this topic has been framed in terms of the related concept of classroom goal structures (CGSs), defined by the kind of motivational goal that teachers primarily emphasize through explicit messages, which constitute one of the features of CMC (Meece et al., 2006; Midgley et al., 2000). The main assessment instruments used in these studies were the scales developed by Midgley et al. (2000). These scales assessed CGS based on students’ perceptions of the emphasis their teachers placed, mainly through explicit messages, on: (a) effort and understanding (mastery goal structure); (b) getting right answers, high scores on tests and good grades (performance-approach structure); and (c) avoiding mistakes in front of others and not doing worse than others (performance-avoidance structure). Subsequent meta-analytic evidence has shown systematic associations between perceived classroom-goal structures and the corresponding personal achievement goals (Bardach et al., 2020a). However, these scales do not assess other specific teaching patterns, beyond teachers’ messages, that contribute to CMC and so they may need to be modified if such a climate is not adequate for fostering learning motivation and associated motivational variables, including interest, effort, self-regulation, perceived ability and, in the present case, resilience. Moreover, they might not be sensitive enough to identify improvements in classroom practices following educational interventions.

**Figure 1:**
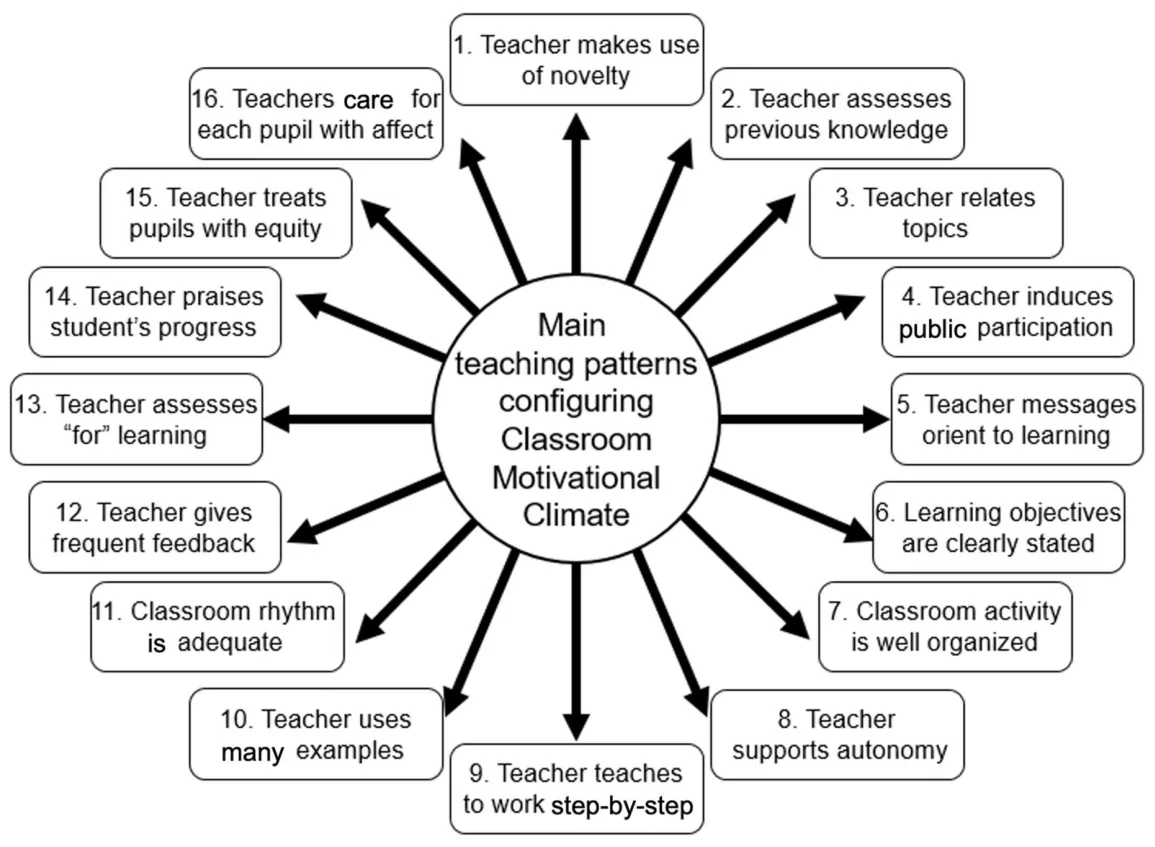
Teaching patterns of Classroom Motivational Climate (CMC) assessed by the Classroom Motivational Climate Questionnaire (CMCQ).

For this reason, Alonso-Tapia and Pardo (2006) revised and summarized the main teaching patterns that, according to different authors, teachers use along the learning sequence, and analyzed the particular effectiveness of each pattern for enhancing learning motivation. Thereafter, considering that CMC results from the particular configuration of such teaching patterns, Alonso-Tapia and Fernández-Heredia (2008) developed the Classroom Motivational Climate Questionnaire (CMCQ). This instrument assesses the extent to which students perceive that a teacher uses the teaching patterns or strategies shown in Figure 1. The total scale score operationalizes perceived CMC by indicating the extent to which the classroom climate is perceived as learning-oriented.

Validation studies have supported the reliability and validity of the CMCQ in different populations and cultural contexts (Alonso-Tapia & Fernández-Heredia, 2008; Alonso-Tapia & Moral, 2010; Villasana & Alonso-Tapia, 2015). Research using the CMCQ has also shown that students who perceive a more learning-oriented CMC report greater teacher-attributed improvements in interest, perceived ability, disposition to effort, and success expectancies, as well as greater satisfaction with their teacher’s work (Alonso-Tapia & Fernández-Heredia, 2008). Additional research has linked a learning-oriented CMC to self-regulation and to perceived change in self-regulation attributed to their teachers’ work (Alonso-Tapia et al., 2014). CMC has also been examined as a protective factor related to subjective resilience (Alonso-Tapia et al., 2013). A later multilevel study found that teachers’ motivational quality was indirectly related to between-classroom differences in CMC through students’ goals and expectancies, and to students’ attribution of motivational improvement through these variables and CMC (Alonso-Tapia et al., 2020).

Recent convergent evidence also supports the relevance of specific teaching practices conceptually related to several components of a learning-oriented CMC. Teacher-provided classroom structure is positively associated with engagement, competence beliefs, and achievement. Interventions aimed at improving such structure showed positive effects on engagement and achievement and negative effects on disengagement (Patall et al., 2024). Likewise, an intervention focused on autonomy-supportive teaching increased autonomy satisfaction and agentic engagement while reducing autonomy dissatisfaction and agentic disengagement (Reeve et al., 2020). Although these studies do not assess the complete CMC construct or academic resilience as defined in the present study, they provide convergent evidence that modifiable teaching practices may support motivational processes and engagement relevant to adaptive responses to academic difficulties. Related research on teacher support also suggests that instrumental and socio-emotional forms of support may be associated with academic motivational resilience through academic self-efficacy. This is conceptually consistent with the role attributed here to teaching patterns that strengthen students’ perceived competence, strategic focus, and expectations of improvement (Kang et al., 2024).

The variables configuring CMC focus students’ attention on motivational, self-regulatory, relational, and process-oriented aspects of learning. According to A. Martin (2002), Villasana et al. (2016, 2017), Skinner et al. (2020), and the recent evidence reviewed above, these aspects may support adaptive responses to academic adversity. It was therefore hypothesized that the more learning-oriented students perceived the CMC to be, the more strongly they would attribute perceived changes in resilience to their teacher. However, students go to school with their own psychological characteristics, such as subjective resilience and success expectancies, which may be associated with their perception of CMC and its relation to perceived change in resilience.

The present study is part of a broader research project on classroom motivational climate and students’ academic adjustment. Within that broader project, the present manuscript examines a specific and distinct research question: the extent to which perceived classroom motivational climate, subjective resilience, and success expectancies are jointly associated with students’ perceived teacher-related change in academic resilience.

Before describing the empirical study, one final conceptual distinction should be made. According to Robinson (2023), motivational climate theory distinguishes three categories of classroom motivational processes: classroom motivational supports (CMSs), provided through teachers’ patterns of action; classroom motivational climate (CMC), defined as “students’ shared perception of the motivational qualities of their classrooms”; and classroom motivational microclimates (CMMCs), defined as “individual perceptions of classroom motivational climate” (p. 92). CMMC and CMC, both often referred to as CMC, are associated with students’ motivation, learning, self-regulation, and coping processes, as shown in multilevel studies comparing their relative predictive capacity (Alonso-Tapia et al., 2020), and may also be associated with perceived change in resilience. In the present study, the focus is not on CMC as a shared classroom perception, but on students’ individual perceptions of CMC and their possible association with perceived change in resilience.

## 2. Materials and Methods

### 2.1. Participants

Participants were 749 students enrolled in secondary (*n* = 185), upper-secondary (*n* = 525), and vocational education programs (*n* = 39) at a single private urban school in France that agreed to participate in the study and served a predominantly middle- to upper-middle-socioeconomic-status student population. They were recruited using a non-probability convenience sampling procedure. The final analytic sample included students aged 14 to 23 years (M = 17.09, SD = 1.59), approximately two-thirds of whom were female (*n* = 496, 66.2%). They belonged to one of thirty-nine classes with a mean of 18.5 students per class. The percentage of males per group ranged from 7% to 80% (Mean: 34%), and the percentage of females, from 20% to 93% (Mean: 66%). Each of the 39 teachers taught one of 19 different subjects. The thirty-nine classes comprised nine secondary education classes, twenty-seven upper-secondary education classes (thirteen in Course 1 and fourteen in Course 2), and three vocational education classes. A complete description of the sample by educational level, course, class group, and gender is provided in Appendix A (Table A1).

### 2.2. Materials

The following instruments were used to test the hypotheses. Representative items from each measure are provided in Appendix A (Table A2).

Classroom Motivational Climate Questionnaire, CMCQ (Alonso-Tapia & Fernández-Heredia, 2008). The CMCQ assesses students’ perceptions of the extent to which their teacher uses teaching patterns that characterize a learning-oriented classroom motivational climate. The questionnaire includes 32 items assessing 16 teaching patterns. Items were rated on a five-point scale ranging from 1 (I totally disagree) to 5 (I totally agree). To reduce acquiescence bias, each teaching pattern was assessed with one positively and one negatively worded item. The questionnaire was translated into French and back-translated into Spanish to assess translation accuracy. Previous validation studies reported satisfactory psychometric properties, with reliability coefficients ranging from 0.92 to 0.93. The psychometric properties and cross-cultural validity of the French version used here were examined by Villasana and Alonso-Tapia (2015). The questionnaire has been previously described in validation studies.

Subjective Resilience Questionnaire, SRQ (Alonso-Tapia et al., 2013). The SRQ assesses students’ perceived resilience when facing adverse events in their relationships with teachers, peers, and family. The cross-cultural validity of the SRQ was examined by Alonso-Tapia and Villasana (2014). It includes a general scale (SR) and three specific scales referring to resilience in relation to teachers (RT), peers (RP), and family or parents (RF). Items were answered on a five-point Likert scale ranging from 1 (I totally disagree) to 5 (I totally agree). Previous research reported McDonald’s omega coefficients (McDonald, 1999) of 0.97 for the general scale, 0.98 for RT, 0.93 for RP, and 0.93 for RF.

Attribution of Perceived Change in Resilience to Teacher Work, APCRT (Alonso-Tapia et al., 2013). This scale assesses the extent to which students attribute perceived changes in resilience to their teacher’s work. This measure captures students’ subjective attribution and does not assess longitudinal change through repeated measurements. It includes eight items answered using a five-point Likert scale ranging from 1 (I totally disagree) to 5 (I totally agree). Previous research reported a reliability coefficient ω = 0.93.

Success Expectancy Scale, SEXP (Alonso-Tapia & Pardo, 2006). This scale is an abbreviated form of the Expectancy Scale and assesses students’ expectations of academic success. It includes ten items, five assessing success expectancies based on perceived self-efficacy or ability (SEF) and five assessing expectancies based on perceived control through effort (CONT). Items were answered on a five-point Likert scale ranging from 1 (I totally disagree) to 5 (I totally agree). Previous research reported a reliability coefficient ω = 0.96 for the general scale.

### 2.3. Procedure

The study was approved by the Research Ethics Committee of the Universidad Autónoma de Madrid, Spain. Participation was voluntary and anonymous. Data were collected electronically. The electronic questionnaire required a response to each item before submission; consequently, there were no item-level missing data in the analytic dataset. Students completed the questionnaires in two 50-min sessions, organized according to their class group and educational level. One of the researchers was present during data collection, provided standardized instructions, and asked students to answer with reference to a specific teacher and subject. The same teacher served as the referent for both the CMCQ and the APCRT.

### 2.4. Data Analysis

Prior to the main analyses, descriptive statistics, correlations, reliability, and discriminant validity were examined for the study variables. Reliability indexes were calculated using Cronbach-α. As for discriminant validity, after calculating the Average Variance Extracted, the Fornell–Larcker criterion was used (Fornell & Larcker, 1981).

**Figure 2:**
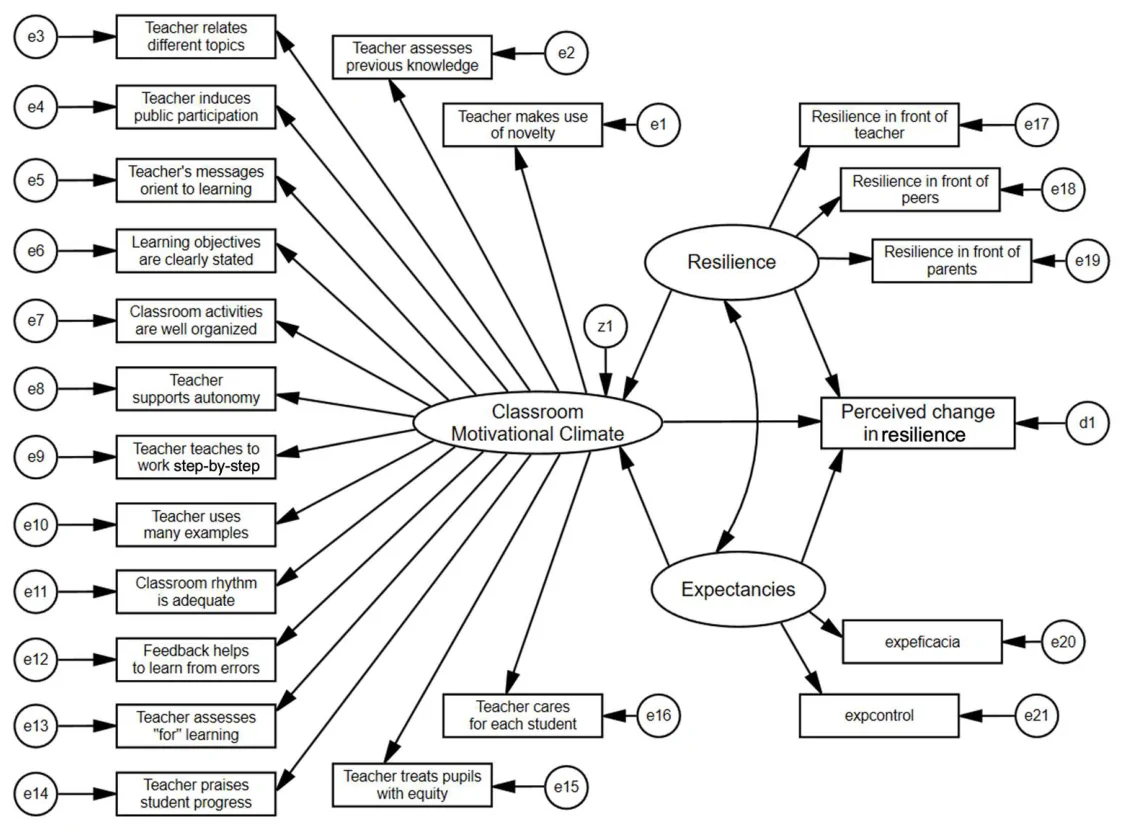
Proposed path analysis with latent variables (PALV-1) model of the associations among subjective resilience, success expectancies, classroom motivational climate, and perceived teacher-related change in academic resilience.

A path analysis with latent variables (PALVs) was conducted using AMOS 22 to examine the extent to which subjective resilience, classroom motivational climate (CMC), and success expectancies were associated with students’ perceived change in resilience attributed to their teacher’s work. The sample was randomly divided into two subsamples of similar size. The first subsample was used to estimate the initial model and to assess the relations among subjective resilience, CMC, success expectancies, and perceived change in resilience within the single model shown in Figure 2 (PALV-1).

Parameters were estimated using the maximum likelihood method after assessing the suitability of the data for this estimation method. Mardia’s coefficient was 21.82, below the reference value of 70 adopted for this analysis (Rodríguez & Ruiz, 2008). Total, direct, and indirect effects were calculated. Model fit was assessed using the chi-square statistic (χ^2^), the χ^2^/df ratio, the goodness-of-fit index (GFI), the incremental fit index (IFI), the comparative fit index (CFI), and the root mean square error of approximation (RMSEA). The interpretation of model fit followed the criteria described by Hair et al. (2010).

In order to cross-validate the results obtained in the initial analysis, a multi-group analysis (PALV-2) was subsequently conducted across the two subsamples. The same estimation method and model-fit criteria used in PALV-1 were applied in the cross-validation analysis.

## 3. Results

### 3.1. Descriptive Analysis, Correlations Between Variables, and Discriminant Validity

Table 1 shows the descriptive statistics of the main variables in the study, their Cronbach-α reliability indexes (in the diagonal), and the correlations between them. All correlations are significant, and Cronbach-α indexes are good and similar to those found in previous studies.

Table 2 shows the average variance extracted of each construct (AVE) and the square of the correlations with the remaining constructs. As can be seen, in all cases AVE is higher than the square of the correlations, a fact showing good discriminant validity. Therefore, the constructs are different, both on conceptual and empirical bases.

### 3.2. PALV Analysis

**Figure 3:**
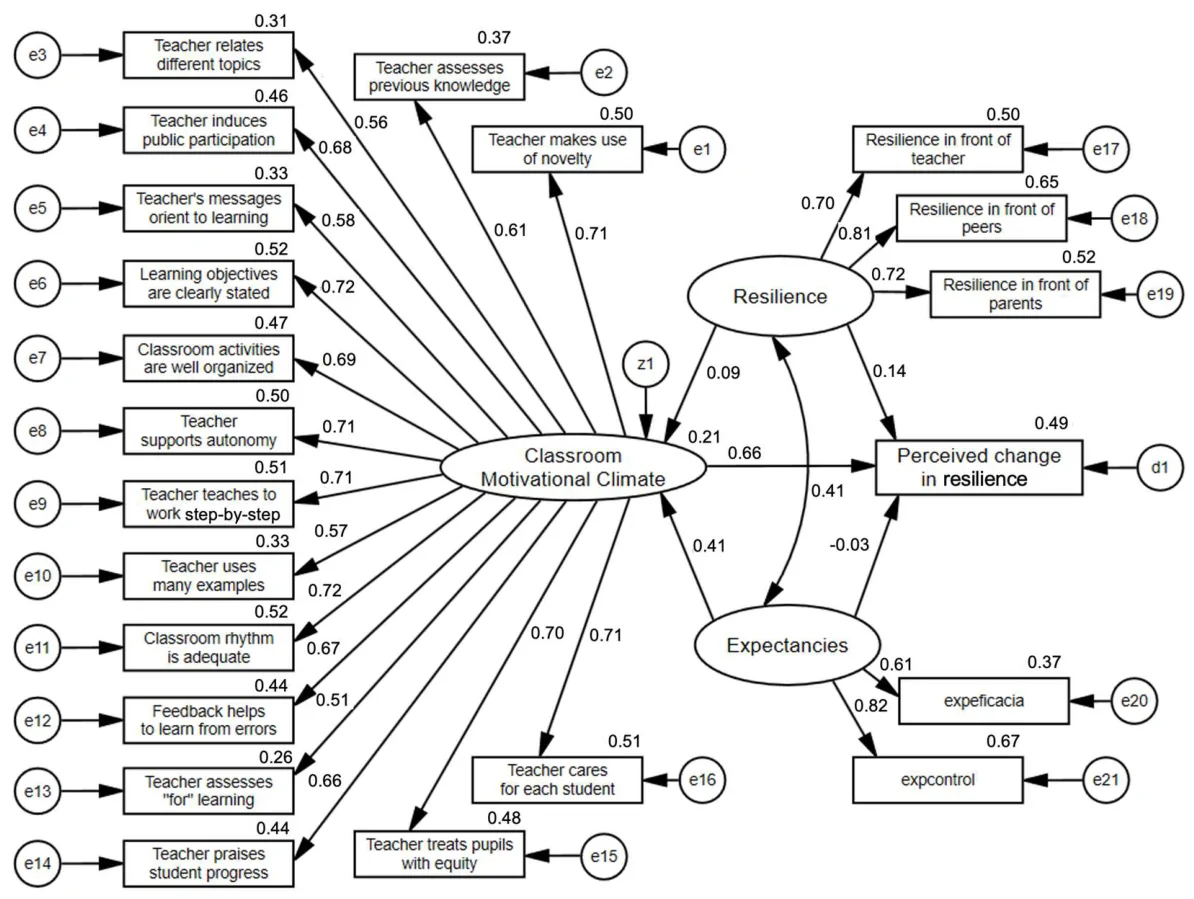
Path Analysis with Latent Variables (PALVs): Associations among the study variables and perceived teacher-related change in academic resilience.

Table 3 shows the standardized total, direct, and indirect effects, and Figure 3, the standardized direct estimates of the PALV model, as well as the squared multiple correlations. All factor loadings (λ) were significant (*p* < 0.001). However, the main focus of this analysis was on the γ regression coefficients assessing the relations between resilience, classroom motivational climate (CMC), and expectancies, and the attribution of perceived change in resilience to the work.

The association between CMC and the attribution of perceived change in resilience to teacher’s work was statistically significant (*p* < 0.001), as expected. CMC showed the strongest direct association with perceived change in resilience (β = 0.66), and the model explained 49% of the variance in the outcome. The model also explained 21% of the variance in perceived CMC. The relation between subjective resilience and perceived change in resilience was also significant (β = 0.14, *p* = 0.004), although its size was small. The model indicated a small indirect association of 0.06 between subjective resilience and perceived change in resilience through CMC. Success expectancies were significantly associated with CMC (*p* < 0.001), and the model indicated an indirect association of 0.27 with perceived change in resilience through CMC. Nevertheless, the direct weight from success expectancies to perceived change in resilience was non-significant.

Table 4 shows the fit statistics of the proposed model (PALV-1). Although the chi-square statistic was significant, χ^2^(204) = 403.55, *p* < 0.001, the χ^2^/df ratio was below the recommended threshold, χ^2^/df = 1.97, and the remaining fit indexes supported an acceptable fit of the model: GFI = 0.95, IFI = 0.94, CFI = 0.94, and RMSEA = 0.05.

The fit statistics for PALV-2 were very similar to those obtained for PALV-1. Although the chi-square statistic was significant, χ^2^(479) = 862.19, *p* < 0.001, the χ^2^/df ratio was also acceptable, χ^2^/df = 1.80, and the remaining fit indices indicated adequate model fit: GFI = 0.95, IFI = 0.94, CFI = 0.94, and RMSEA = 0.03.

In addition, the model comparison statistics against the unconstrained model showed that imposing equality constraints on measurement weights (*p* = 0.225), structural weights (*p* = 0.250), structural covariances (*p* = 0.260), structural residuals (*p* = 0.327), and measurement residuals (*p* = 0.359) did not significantly reduce model fit, as shown in Table 5. These results support the stability of the model across the two validation subsamples.

## 4. Discussion

The present findings suggest that students’ perceptions of a learning-oriented classroom motivational climate are strongly associated with the extent to which they attribute perceived positive changes in their academic resilience to their teacher’s work. This result is consistent with motivational approaches that view learning situations as contexts in which students may develop competence, confidence, and more adaptive ways of dealing with difficulty (Dweck & Elliott, 1983; Skinner et al., 2020). Because adolescents may experience academic adversity in interactions with teachers, peers, and family members, different forms of support may be needed depending on the source of adversity.

Accordingly, the objective of this study was to examine students’ perceptions of teachers’ contributions to perceived changes in academic resilience. Specifically, it examined the extent to which CMC, students’ success expectancies, and subjective resilience were related to perceived change in resilience. The starting point was to consider whether subjective resilience and success expectancies were related to perceived CMC and whether perceived CMC, in turn, was related to students’ attribution of perceived change in resilience to their teacher’s work. The results yielded three main findings.

First, the findings showed that subjective resilience was virtually unrelated to the perception of the classroom climate as learning-oriented. Its direct association with students’ attribution of perceived change in resilience was statistically significant but very small, explaining only 2.5% of the variance. Thus, students who reported higher subjective resilience did not necessarily perceive the classroom climate as more learning-oriented than their peers did.

A second finding concerned the relationship among success expectancies, CMC, and academic resilience. The importance of expectations is well established in contemporary motivation research. Situated expectancy-value theory identifies expectations for success as central motivational beliefs related to students’ engagement, performance, and achievement-related choices, while emphasizing that such beliefs are shaped by social and educational contexts (Eccles & Wigfield, 2020). In line with this, the present study showed that success expectancies were directly related to perceived CMC, although they were not directly related to students’ attribution of perceived change in academic resilience to their teacher’s work. This does not imply that success expectancies are unrelated to perceived change in resilience. Rather, the pattern of results is consistent with an indirect association through perceived CMC: students with higher success expectancies tended to perceive the classroom climate as more learning-oriented.

Third, the central finding was that students’ attribution of perceived change in resilience to their teacher’s work was most strongly associated with perceived CMC. This finding concerns the extent to which students who perceived changes in their resilience attributed those changes to their teacher’s work. The model explained 49% of the variance in perceived teacher-related change in academic resilience. Within this model, perceived CMC showed the strongest direct association with perceived teacher-related change in academic resilience (β = 0.66). This result indicates a strong direct association between perceived CMC and students’ attribution of perceived positive changes in resilience to their teacher’s work, beyond the contribution of success expectancies and subjective resilience, whose indirect effects are conveyed through the CMC. This central role of CMC is consistent with recent research highlighting classroom climate as a relevant contextual source of students’ motivation and learning (Alonso-Tapia et al., 2020; Alonso-Tapia & Ruiz-Díaz, 2025; Robinson, 2023).

This central finding has practical implications. The strong association between perceived CMC and students’ attribution of perceived positive changes in resilience to their teacher’s work is educationally relevant. This finding suggests that teaching practices may be relevant to students’ perceived capacity to manage and recover from academic adversity. Regardless of their subjective resilience, students who perceived the climate as learning-oriented tended to report greater perceived positive changes in their resilience. This practical implication is consistent with intervention evidence showing that teachers can learn to adopt more autonomy-supportive instructional practices and that these practices benefit students’ motivation and classroom functioning (Reeve & Cheon, 2021).

At the same time, these implications should be interpreted cautiously because the present study did not test an intervention. Nevertheless, they are consistent with recent evidence indicating that school-based interventions can enhance resilience in children and adolescents, although the average effect is small and heterogeneous (Cai et al., 2025). They also converge with studies of school-based group mentoring showing that relational resources within school contexts may promote resilience among vulnerable high-school students and may be associated with academic outcomes (Chan et al., 2020; Kuperminc et al., 2020).

The present study contributes to understanding the motivational factors associated with students’ attribution of perceived change in academic resilience. However, there are limitations that provide direction for further research. First, the cross-sectional design does not allow causal conclusions or the demonstration of actual change over time. Although students reported perceived changes in their resilience and attributed them to their teacher’s work, resilience was not assessed repeatedly over time. The findings therefore concern students’ perceived change in academic resilience and their attribution of that change to the teacher.

In addition, the sample included students from secondary, upper-secondary, and vocational education, with an age range extending from adolescence into young adulthood. The convenience sampling procedure also limits the generalizability of the findings. Future studies should examine whether the same pattern of associations holds across educational stages and tracks. Because several measures were originally validated primarily with younger students in secondary and upper-secondary education—though also with university students (Abelló et al., 2021)—their measurement equivalence across this broader age range should also be examined.

Second, perceived CMC, success expectancies, subjective resilience, and perceived change in resilience attributed to teacher’s work were all assessed through students’ self-reports. Moreover, both the CMCQ and the measure of perceived change referred to the same teacher. Consequently, the magnitude of the associations may have been influenced by common-source, common-method, and common-referent variance, including a possible halo effect. Future studies should incorporate repeated measures, classroom observations, and information provided by teachers or other observers.

Third, classroom climate includes not only motivational climate but also emotional climate, social climate, and discipline-management climate (Alonso-Tapia & Ruiz-Díaz, 2025). These facets are related and may also contribute to students’ perceived change in resilience. The fact that one of the teaching patterns assessed by the CMCQ is “dedication to each student” supports this interpretation. Therefore, further research on the relationship between the affective and social quality of teacher–student interactions and resilience is needed. This extension seems especially relevant because recent evidence in higher education suggests that teacher emotional support may be associated with academic self-efficacy, academic resilience, and engagement (Guo et al., 2025). Although this evidence comes from a different educational level, it reinforces the need to examine affective and social dimensions of teacher–student interaction alongside motivational climate.

Moreover, classroom climate can be considered both an individual perception and a characteristic shared by students within the same classroom. Because this nesting was not modeled, standard errors may have been underestimated, and significance tests may therefore be overly optimistic. Future studies should therefore account for the nesting of students within classrooms, teachers, and schools through multilevel designs (Bardach et al., 2020b; Alonso-Tapia & Ruiz-Díaz, 2025). This recommendation is also consistent with research conceptualizing safe and supportive schools as multidimensional environments encompassing connectedness, emotional safety, engagement, norms, and policies (Terrell et al., 2020). It is also consistent with recent work treating school climate as a component of school capacity that may require aggregation beyond individual perceptions (Terrell et al., 2025).

Finally, academic resilience has been conceptualized and measured in different ways (Rudd et al., 2021; Ye et al., 2024). The present study adopts a subjective, scale-based operationalization of academic resilience, which should be distinguished from approaches that infer resilience from positive academic achievement under objectively assessed conditions of adversity. Longitudinal, multilevel, and multi-informant studies are therefore needed to examine whether changes in CMC precede actual changes in academic resilience and whether the findings generalize to other educational and cultural contexts.

## 5. Conclusions

In conclusion, the present study suggests that students’ perceptions of a learning-oriented classroom motivational climate are strongly associated with the extent to which they attribute perceived positive changes in academic resilience to their teacher’s work. This association remained relevant after accounting for subjective resilience and success expectancies. Although the cross-sectional, self-report design precludes causal conclusions, the findings support the educational relevance of classroom motivational climate as a potentially modifiable contextual factor linked to students’ perceived teacher-related change in academic resilience. Future longitudinal, multilevel, and multi-informant studies are needed to examine whether changes in teaching practices precede actual changes in academic resilience across educational stages and cultural contexts.

## Figures and Tables

**Table 1:** Descriptive statistics, and Cronbach-α reliability indexes (in the diagonal) of the main variables in the study, and correlations between them ^1^.

	M	SD	RES	EXP	CMC	C-RES
RES	100.07	14.07	0.84	0.31 ***	0.19 ***	0.20 ***
EXP	35.99	5.68		0.70	0.32 ***	0.18 ***
CMC	117.54	18.80			0.92	0.64 ***
C-RES	23.53	5.67				0.87

^1^ RES: Resilience; EXP: Expectancies; CMC: Classroom Motivational Climate; C-RES: Criterion of Change in Resilience; *** *p* < 0.001.

**Table 2:** Average variance extracted, square correlations between variables in the study and discriminant validity following the Fornell–Larcker criterion ^1^.

	AVE	R^2^ with EXP	R^2^ with CMC	R^2^ with C-RES	DV
RES	0.55	0.10	0.04	0.04	+/+/+
EXP	0.52		0.10	0.03	+/+
CMC	0.43			0.40	+
C-RES	0.44				

^1^ AVE: Average Variance Extracted; RES: Resilience; EXP: Expectancies; CMC: Classroom Motivational Climate; C-RES: Criterion of Change in Resilience; DV: Discriminant Validity; +: Discriminant validity supported according to the Fornell–Larcker criterion (AVE greater than the squared interconstruct correlation).

**Table 3:** Model-implied standardized total, direct, and indirect effects of the study variables on perceived change in resilience.

	Predictors		
Standardized Effects	Resilience	Expectancies	CMC ^1^
Total effects	0.20	0.24	0.66
Direct effects	0.14	−0.03	0.66
Indirect effects	0.06	0.27	0

^1^ CMC: Classroom Motivational Climate.

**Table 4:** Goodness-of-fit statistics for path analyses with latent variables (PALVs) from CMC, success expectancies, and resilience to attribution of perceived change in resilience attributed to teacher’s work.

	χ^2^	df	*p*	χ^2^/df	GFI	IFI	CFI	RMSEA
PALV-1 (N = 375)	403.55	204	<0.001	1.97	0.95	0.94	0.94	0.05
PALV-2 CVA ^1^ (N = 375/374)	862.19	479	<0.001	1.80	0.95	0.94	0.94	0.03

^1^ CVA: Cross-Validation Analysis.

**Table 5:** Cross-validation of the model using multi-group analysis with two samples. Chi-square difference tests comparing each constrained model with the unconstrained multi-group model.

Analysis	Model	df	χ^2^	*p*
PALV-2: CVA	Invariance of Measurement weights	21	25.52	0.225
	Invariance of Structural weights	43	48.82	0.250
	Invariance of Structural covariances	45	50.66	0.260
	Invariance of Structural residuals	48	51.83	0.327
	Invariance of Measurement residuals	49	51.97	0.359

## Data Availability

The data presented in this study are available on request from the corresponding author due to privacy and ethical restrictions.

## Abbreviations

The following abbreviations are used in this manuscript:

CMC	Classroom Motivational Climate
CMS	Classroom Motivational Supports
CMMC	Classroom Motivational Microclimates
PALV	Path Analysis with Latent Variables
TARGET	Task, Authority, Recognition, Grouping, Evaluation, and Time
CGS	Classroom Goal Structures
CMCQ	Classroom Motivational Climate Questionnaire
SRQ	Subjective Resilience Questionnaire
SR	General Scale
RT	Resilience in Relation to Teachers
RP	Resilience in Relation to Peers
RF	Resilience in Relation to Family or Parents
APCRT	Attribution of Perceived Change in Resilience to Teacher Work
SEXP	Success Expectancy Scale
SEF	Self-efficacy
CONT	Perceived Control Through Effort

## Appendix A

**Table A1:** Sample description by educational level, course, class group, and gender.

Secondary Education (Only one course). Age: M = 16.00, SD = 1.08															
Group	1	2	3	4	5	6	7	8	9						Total
Males	8	2	4	3	18	8	11	4	1						59
Females	27	27	9	14	7	12	3	13	18						126
Upper Secondary, Course 1. Age: M = 16.67, SD = 1.16															
Group	10	11	12	13	14	15	16	17	18	19	20	21	22		Total
Males	2	4	5	4	15	6	0	13	14	0	0	7	6		76
Females	12	12	25	4	11	8	17	3	11	29	27	2	8		169
Upper Secondary, Course 2. Age: M = 17.80, SD = 1.60															
Group	23	24	25	26	27	28	29	30	31	32	33	34	35	36	Total
Males	7	4	8	9	16	1	3	0	15	11	13	12	2	1	92
Females	23	34	7	7	14	13	10	15	13	12	3	27	12	8	188
Vocational Education (Only one course). Age: M = 19.64, SD = 1.51															
Group	37	38	39												Total
Males	8	10	8												26
Females	8	3	2												13

**Table A2:** Example items from the measures used in the study.

**Measure**	**Subscale/Indicator**	**Example Item(s)**
Classroom Motivational Climate Questionnaire (CMCQ)	—	“This teacher praises our effort to learn whenever possible.” “This teacher does not allow the freedom of choosing how to work or with whom.”
Subjective Resilience Questionnaire (SRQ)	Resilience in Relation to Teachers (RT)	“Although a teacher doesn’t devote time to answer my questions or pay some attention to me when I’m faced with a difficulty, I usually do my best to learn.”
	Resilience in Relation to Peers (RP)	“If my classmates don’t take me into consideration when they organise some event, I don’t get too worried because I find other things to do.”
	Resilience in Relation to Family or Parents (RF)	“Although my parents don’t devote time to listen to me when I need their help, I don’t let difficulties discourage me.”
Attribution of Perceived Change in Resilience to Teacher Work (APCRT)	—	“The way in which this teacher helps me face difficulties makes me get less and less discouraged when I have a failure in my studies.” “If my classmates sometimes ignore me or try to hurt me, I don’t get discouraged and know what to do due to this teacher’s work.”
Success Expectancy Scale (SEXP)	Perceived Control Through Effort (CONT)	“I expect to achieve good results in my studies thanks to my effort and dedication.”
	Perceived Self-Efficacy/Ability (SEF)	“I am not particularly hard-working, and even if I make an effort, I do not think I will achieve good results in my studies.”
